# Monocyte-to-lymphocyte ratio is associated with 28-day mortality in patients with acute respiratory distress syndrome: a retrospective study

**DOI:** 10.1186/s40560-021-00564-6

**Published:** 2021-08-06

**Authors:** Lijuan Yang, Chang Gao, Fengyuan Li, Ling Yang, Jiahao Chen, Shiqi Guo, Ying He, Qiang Guo

**Affiliations:** 1 Department of Critical Care Medicine, The First Affiliated Hospital of Soochow University, Suzhou, Jiangsu China; 2 Department of Critical Care Medicine, Suzhou Dushuhu Public Hospital (Dushuhu Public Hospital Affiliated to Soochow University, Medical Center of Soochow University), Suzhou, Jiangsu China; 3 Pneumology Department, Department of Emergency, Department of Critical Care Medicine, Suzhou Dushuhu Public Hospital (Dushuhu Public Hospital Affiliated to Soochow University, Medical Center of Soochow University), The First Affiliated Hospital of Soochow University, No.9 Chongwen Road, Suzhou Industrial Park, Suzhou, Jiangsu China

**Keywords:** Monocytes, Lymphocytes, Prognostic, Acute respiratory failure, Acute lung injury, Mortality

## Abstract

**Background:**

Systemic inflammation relates to the initiation and progression of acute respiratory distress syndrome (ARDS). Neutrophil-to-lymphocyte ratio (NLR) and red blood cell distribution width (RDW)/albumin ratio have been reported to be predictive prognostic biomarkers in ARDS patients. However, the role of monocyte-to-lymphocyte ratio (MLR) as a prognostic inflammatory biomarker in a variety of diseases is rarely mentioned in ARDS. In this study, we explored the relationship between MLR and disease severity in ARDS patients and compared it with other indicators associated with 28-day mortality in patients with ARDS.

**Methods:**

We retrospectively included 268 patients who fulfilled the Berlin definition of ARDS and were admitted to a single institute from 2016 to 2020. Clinical characteristics and experimental test data were collected from medical records within 24 h after the ARDS diagnosis. MLR, NLR, and RDW/albumin ratio levels were calculated. The primary clinical outcome was 28-day mortality. Logistic regression analysis was used to illustrate the relationship between indicators and 28-day mortality. Receiver operating characteristic (ROC) curve was used to evaluate the area under the curve (AUC), and propensity score matching (PSM) was employed to validate our findings.

**Results:**

The median MLR values were higher for non-survivors than for survivors before and after matching (*P*<0.001, *P*=0.001, respectively). MLR values were significantly associated with 28-day mortality (OR 2.956; 95% CI 1.873–4.665; *P*<0.001). MLR and NLR indicators were combined for predictive efficacy analysis, and its AUC reached 0.750. There was a significant increase in 28-day mortality depending on the increasing MLR level: low MLR group 38 (20.4%), high MLR group 47 (57.3%) (*P*<0.001).

**Conclusions:**

Higher MLR values were associated with 28-day mortality in patients with ARDS. Further investigation is required to verify this relationship with prospectively collected data.

**Supplementary Information:**

The online version contains supplementary material available at 10.1186/s40560-021-00564-6.

## Introduction

Acute respiratory distress syndrome (ARDS) is a non-cardiogenic pulmonary edema induced by lung damage caused by inflammation, leading to fatal respiratory failure [1, 2]. Despite recent advances in intensive care models [3, 4], mortality in ARDS patients remains high. A multicenter observational cohort study reported that the prevalence of ARDS was 10.4% at ICU admission and that its overall hospital mortality was 40.0% [5]. An effective marker for prognosis in ARDS is particularly important considering that ARDS appears to be under-recognized and under-treated and remains a major challenge to critical care medicine. However, the Acute Physiology and Chronic Health Evaluation II (APACHE II) score, Sequential Organ Failure Assessment (SOFA) score, and Simplified Acute Physiology Score (SAPS), which are used to assess the prognosis of critically ill patients, are not specific to ARDS. The application of risk prediction models for ARDS patients requires many variables and complex formulas [6]. In addition, several studies have analyzed biomarkers in patients with ARDS, such as interleukin (IL)-1 beta, IL-6 [7], mucins with selectin ligands [8], and Th17/Treg ratio [9]. However, those parameters cannot be detected immediately at bedside; most require special biological samples from patients and integration with other clinical data, which prolongs the diagnostic process.

Clinical and animal studies have shown that the activation of multiple inflammatory cells and the release of inflammatory mediators contribute to the development and progression of ARDS [10], and the associations between ARDS and inflammatory biomarkers, such as IL-18, red blood cell distribution width (RDW)/albumin ratio, and neutrophil-to-lymphocyte ratio (NLR), have been explored [11–13]. Monocyte-to-lymphocyte ratio (MLR) is the absolute monocyte count divided by the absolute lymphocyte count and has been demonstrated to be a novel hematological and inflammatory parameter. The clinical utility of the MLR as a combined index has not yet been evaluated in patients with ARDS. Based on previous studies, we propose the hypothesis that MLR may be associated with disease severity and mortality in ARDS. In this study, we sought to investigate the association between MLR and 28-day mortality in patients with ARDS and compare it with other indicators.

## Materials and methods

### Study design and patient population

**Fig. 1:**
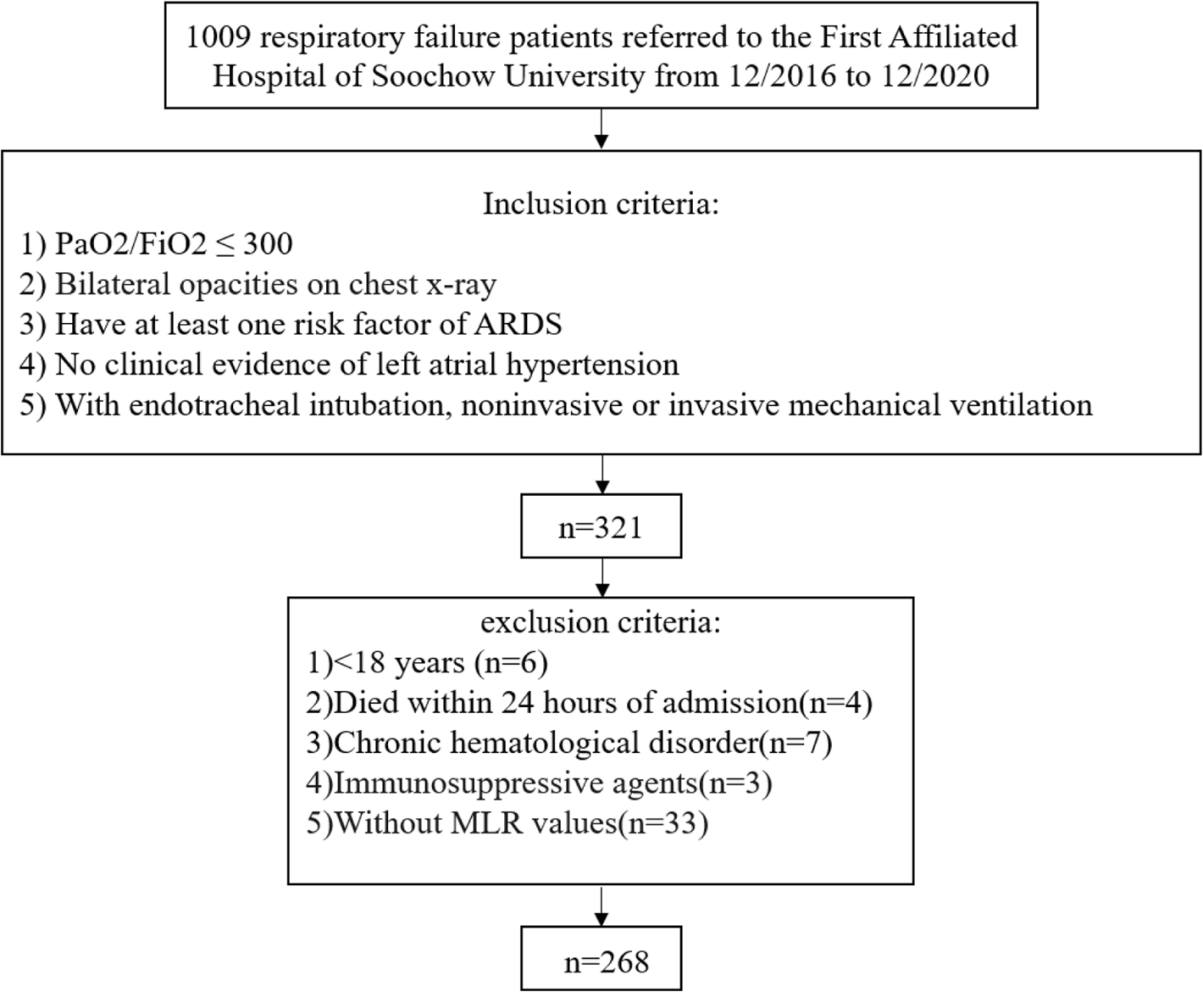
Research flowchart. ARDS, acute respiratory distress syndrome; MLR, monocyte-to-lymphocyte ratio

From December 2016 to December 2020, 1009 patients were diagnosed with respiratory failure in the First Affiliated Hospital of Soochow University. Two medical doctors in the Department of Critical Care Medicine at First Affiliated Hospital of Soochow University reviewed the medical records of all patients. A total of 268 patients who meet the inclusion criteria and none of the exclusion criteria were retrospectively enrolled in the study (Fig. 1). All eligible patients met the Berlin definition criteria for ARDS [2] and had monocyte and lymphocyte count results within 24 h after the ARDS diagnosis. Patients who were less than 18 years old, died within 24 h of admission, or were with chronic hematological disorder were excluded. Institutional approval was provided by the Clinical Research Ethics Committee of First Affiliated Hospital of Soochow University (Jiangsu, China). Written informed consent was waived due to the retrospective nature of the study. All patient information was recorded anonymously to ensure confidentiality.

### Data extraction

Clinical data of all eligible patients were collected through the medical record system of our hospital, including baseline demographic information, past medical history, the risk factors of ARDS, and types of infection. Laboratory test results include the following: PaO2/FiO2, white blood cell (WBC) counts, hemoglobin (Hb), red cell distribution width (RDW), neutrophil counts, lymphocyte counts, monocyte counts, lactate, albumin, aspartate aminotransferase (AST), alanine aminotransferase (ALT), creatinine (Cr), and blood urea nitrogen (BUN). SOFA and APACHE II scores were used to assess the severity of the patients’ general condition. These clinical data were recorded within 24 h after the ARDS diagnosis. In addition, we also recorded the monocyte counts, neutrophil counts, and lymphocyte counts on the third day and the fifth day. MLR, NLR, and RDW/albumin were calculated. Two independent authors completed the data collection. All patients were followed up for 28 days. The primary clinical outcome was 28-day mortality. The interventions, duration of ventilation, and hospital length of stay were also recorded. Steroid therapy was defined as at least a dose (≥ 0.5mg/kg) of methylprednisolone during hospitalization [14, 15].

### Statistical analysis

Comparisons between continuous variables were analyzed using *t*-test or Mann-Whitney *U* test based on variable distribution, presented as mean ± standard deviation or medians (quartiles). Categorical variables were compared using chi-square test or Fisher’s exact test and denoted as relative frequencies and percentages. In order to compare whether there was a difference in MLR values between the survivors and non-survivors, a 1:1 propensity score matching (PSM) was used to minimize the imbalance of baseline characteristics (including age, sex, previous history, risk factors of ARDS, types of infection, PaO2/FiO2, categories of ARDS, laboratory tests, APACHE II score, SOFA score, and interventions) between the two groups. A multivariate logistic regression analysis model was used to assess the patient’s propensity scores, with a caliper width of 0.02 in our study. In the matched data, paired samples *T* test and Wilcoxon signed-rank and McNemar’s tests were used to compare baseline characteristics for continuous variables and proportions, respectively.

Multivariate logistic regression analysis was carried out to identify the independent predictors of 28-day mortality, and these included all of the possible cause variables with *P* values less than 0.10 that were selected from a pre-established list (Table S1) using the forward logistic regression method (entering a variable if *P* values are less than 0.05, removing a variable if *P* values are more than 0.10). Binary logistic regression analysis was used to combine MLR and NLR. The efficiency of predicting 28-day mortality in patients with ARDS was evaluated by receiver operating characteristic (ROC) analysis and reported area under the curve (AUC), and the significant cutoff value, maximum sensitivity, and specificity were determined. Two-tailed *P*-values <0.05 were considered statistically significant. All statistical analyses used the SPSS software version 24.0 (SPSS Inc, Chicago, IL).

### Power analysis

To calculate the sample size for the current research, the power analysis was carried out using Power and Sample Size Calculation version 15.0.5 (Department of Biostatistics, Vanderbilt University School of Medicine, Nashville, TN). We estimated the incidence of high MLR in the survival group would be approximately 17%. The odds ratio (OR) was 3. Such assumption was based on pilot data for 2018–2019. Assuming a 1:2 ratio of patients in the non-survival group and survival group, 73 and 146 patients (total of 219) were required to show an association between high MLR and 28-day mortality in ARDS patients at a two-tailed *α* of 0.05 and power of 0.90. Considered with a certain exclusion rate, we eventually included a total of 268 ARDS patients for 5 years.

## Results

### Baseline patient characteristics

**Fig. 2:**
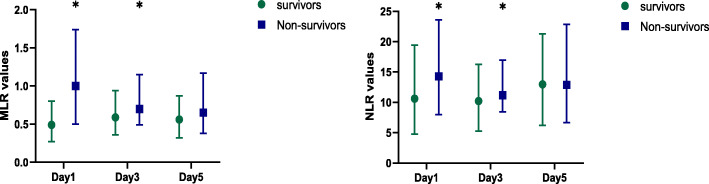
Evolution of MLR and NLR over the first 5 days in patients with ARDS. Values within 24 h, third days, and fifth days after ARDS diagnosis. The median (interquartile range) MLR and NLR values statistically different between survivors and non-survivors in the first and third day: MLR, 0.49 (0.27–0.8), 1 (0.5–1.74), *P*<0.001; 0.59 (0.36–0.94), 0.70 (0.49–1.15), *P*=0.031, respectively; NLR, 10.61 (4.75–19.42), 14.28 (7.97–23.62), *P*=0.003; 10.21 (5.27–16.29), 11.17 (8.45–16.96), *P*=0.027, respectively. ARDS, acute respiratory distress syndrome; MLR, monocyte-to-lymphocyte ratio; NLR, neutrophil-to-lymphocyte ratio. *Values statistically different between non-survivors and survivors

The baseline and clinical characteristics of the survivors and non-survivors are shown in Table 1. Compared with survivors, non-survivors had higher APACHE II scores, and SOFA scores at admission (*P* =0.004, *P*=0.009, respectively) were more likely to use interventions such as albumin infusion, transfusion, and alimentotherapy during hospitalization (*P*=0.001, *P*=0.037, *P*=0.010, respectively) and had shorter hospital length of stay (*P*=0.038). For laboratory parameters, non-survivors had higher MLR and NLR, whereas survivors had higher PaO_2_/FiO_2_ ratio. Furthermore, categories of ARDS in the two groups were significantly different (*P*=0.015). MLR values between the two groups remained different after matching (*P*=0.001). The median (interquartile range) MLR and NLR values were statistically different between survivors and non-survivors in the first and third day: MLR, 0.49 (0.27–0.8), 1 (0.5–1.74), *P*<0.001; 0.59 (0.36–0.94), 0.70 (0.49–1.15), *P*=0.031, respectively; NLR, 10.61 (4.75–19.42), 14.28 (7.97–23.62), *P*=0.003; 10.21 (5.27–16.29), 11.17 (8.45–16.96), *P*=0.027, respectively. The median (interquartile range) MLR and NLR values between survivors and non-survivors were not significantly different on the fifth day: MLR, 0.56 (0.32–0.87), 0.65 (0.38–1.17), *P*=0.148; NLR, 12.97 (6.2–21.28), 12.88 (6.66–22.86), *P*=0.761. The median (interquartile range) MLR and NLR values in the non-survivors showed a decreasing trend after the initial treatment (Fig. 2).

**Table 1:** Comparison of baseline characteristics of unmatched and matched patients according to the survival status

Variables	Original cohort			Matched cohort		
	Survivors (*n*=183)	Non-survivors (*n*=85)	*P* value	Survivors (*n*=56)	Non-survivors (*n*=56)	*P* value
Age (years)	67 (59–72)	67 (63–73)	0.165	67 (60.25–73)	67 (62–73)	0.730
Male, *n* (%)	133 (72.7)	59 (69.4)	0.581	39 (69.6)	39 (69.6)	1.000
Smoking, *n* (%)	60 (32.8)	25 (29.4)	0.581	14 (25)	18 (32.1)	0.557
Alcohol abuse, *n* (%)	45 (24.6)	18 (21.2)	0.540	10 (17.9)	12 (21.4)	0.815
Hypertension, *n* (%)	85 (46.4)	31 (36.5)	0.125	22 (39.3)	24 (42.9)	0.851
Diabetes mellitus, *n* (%)	52 (28.4)	22 (25.9)	0.666	21 (37.5)	17 (30.4)	0.572
Coronary artery disease, *n* (%)	36 (19.7)	18 (21.2)	0.775	12 (21.4)	13 (23.2)	1.000
Risk factor, *n* (%)			0.531			0.912
Pneumonia	167 (91.3)	79 (92.9)		51 (91.1)	51 (91.1)	
Aspiration	8 (4.4)	2 (2.4)		2 (3.6)	1 (1.7)	
Sepsis	3 (1.6)	3 (3.5)		2 (3.6)	3 (5.5)	
Others	5 (2.7)	1 (1.2)		1 (1.7)	1 (1.7)	
Types of infection, *n* (%)			0.941			0.931
Bacteria	139 (76%)	64 (75.3%)		49 (87.6)	49 (87.6)	
Virus	6 (3.3%)	2 (2.4%)		1 (1.7)	1 (1.7)	
Fungus	24 (13.1)	13 (15.3%)		1 (1.7)	2 (3.6)	
Unknown	14 (7.6%)	6 (7 %)		5 (9)	4 (7.1)	
PaO_2_/FiO_2_ (mmHg)	145 (106–208.18)	131 (85.6–177.03)	0.032	144.64±62.79	145.40 ±58.73	0.948
Categories of ARDS, *n* (%)			0.015			0.770
Mild	47 (25.7)	10 (11.8)		12 (21.4)	9 (16.1)	
Moderate	95 (51.9)	46 (54.1)		27 (48.2)	32 (57.1)	
Severe	41 (22.4)	29 (34.1)		17 (30.4)	15 (26.8)	
WBC, 10^9^/L	9.8 (7.02–11.84)	9.22 (6–11.46)	0.249	10.44 (8.55–12.15)	9.62 (7.73–11.44)	0.153
Hemoglobin, g/L	111 (96–127)	111 (94.5–126)	0.460	106 (94–126.75)	112 (99–126.75)	0.884
RDW, %	14 (13–15.3)	13.6 (12.85–14.6)	0.175	13.9 (12.75–15.28)	13.45 (12.8–14.55)	0.964
Lactate, mmol/L	2.2 (1.4–3.7)	2.6 (1.55–4.3)	0.082	2.1 (1.3–3.33)	2.6 (1.5–3.73)	0.152
Albumin, g/L	32.9 (28.9–37.7)	32.30 (28.6–37.5)	0.417	32.89±6.98	32.86±5.86	0.979
MLR	0.49 (0.27–0.8)	1 (0.5–1.74)	<0.001	0.56 (0.34–0.77)	0.83(0.47–1.68)	0.001
NLR	10.61(4.75–19.42)	14.28 (7.97–23.62)	0.003	13.56 (5.98–25.12)	12.96 (7.18–20.75)	0.613
RDW/albumin, %/g/L	0.43 (0.37–0.51)	0.43 (0.37–0.5)	0.914	0.43 (0.36–0.52)	0.43 (0.36–0.49)	0.660
AST	52.2 (20.5–145.2)	71 (28.7–138.3)	0.317	42.35 (20.1–128.25)	88.05 (36.98–147.9)	0.011
ALT	62 (20–126)	40.3 (21.1–133.3)	0.485	44.6 (16.13–103.75)	43.4 (23.03–139.15)	0.324
Cr, μmol/L	100.7 (60.2–183)	124.9 (62–198.15)	0.357	107 (58.18–188.7)	111.3(60.65-192)	0.695
BUN, mmol/L	13.31 (7–18)	13.55 (9.85–19.25)	0.232	13.09±6.45	13.89±7.28	0.512
APACHE II score	12 (9–16)	15 (10–18)	0.004	14.09±4.50	13.66 ±4.57	0.628
SOFA score	7 (6–9)	8 (7–9)	0.009	8 (7–9)	8 (6.25–9)	0.340
Interventions, *n* (%)						
Steroid^a^	121 (66.1)	63 (74.1)	0.189	39 (69.6)	42 (75)	0.690
Hypoglycemic	56 (30.6)	25 (29.4)	0.844	25 (44.6)	19 (33.9)	0.362
Alimentotherapy	113 (61.7)	66 (77.6)	0.010	39 (69.6)	42 (75)	0.607
Albumin infusion	81 (44.3)	56 (66.9)	0.001	36 (64.3)	30 (53.6)	0.286
Transfusion	50 (27.3)	33 (38.8%)	0.037	19 (33.9)	19(33.9)	1.000
CRRT	22 (12)	17 (20)	0.085	8 (14.3)	8 (14.3)	1.000
ECMO	6 (3.3)	5 (5.8)	0.317	1 (1.7)	1 (1.7)	1.000
Mechanical ventilation	108 (59)	51 (60)	0.879	37 (66.1)	35 (62.5)	0.851
Duration of ventilation	7 (2–13)	8 (3.5–13)	0.479	8.95±7.03	9.05±6.43	0.933
Hospital length of stay	20 (12–26)	17 (11–23)	0.038	19.00±9.81	18.30±8.19	0.662

^a^Steroid therapy was defined as at least a dose (≥ 0.5mg/kg) of methylprednisolone during hospitalization

*ARDS*, acute respiratory distress syndrome; *WBC*, white blood cell; *RDW*, red cell distribution width; *MLR*, monocyte-to-lymphocyte ratio; *NLR*, neutrophil-to-lymphocyte ratio; *AST*, aspartate aminotransferase; *ALT*, alanine aminotransferase; *Cr*, creatinine; *BUN*, blood urea nitrogen; *APACHE II*, Acute Physiology and Chronic Health Evaluation II; *SOFA*, Sequential Organ Failure Assessment; *CRRT*, continuous renal replacement therapy; *ECMO*, extracorporeal membrane oxygenation

### Independent predictors for 28-day mortality in ARDS patients

Clinical variables with *P*-values less than 0.1 in univariate logistic regression analysis were included in the multivariate logistic regression analysis to identify the independent predictors for death. The *P*-value for the Hosmer-Lemeshow test was 0.834. MLR (OR 2.956; 95% CI 1.873–4.665; *P*<0.001), NLR (OR 0.972; 95% CI 0.950–0.994; *P*=0.012), APACHE II score (OR 1.088; 95% CI 1.024–1.156; *P*=0.007), SOFA score (OR 1.220; 95% CI 1.066–1.395; *P*=0.004), and alimentotherapy (OR 2.809; 95% CI 1.380–5.719; *P*=0.004) were found to be the independent predictors for 28-day mortality in ARDS patients (Table 2). In the matched cohort, the multivariate logistic regression analysis showed that MLR (OR 2.062; 95% CI 1.198–3.550; *P*=0.009) was the independent predictor for 28-day mortality.

**Table 2:** Univariate and multivariate logistic regression analysis of 28-day mortality prediction for patients with ARDS

Variables	Univariate analysis		Multivariate analysis	
	OR (95% CI)	*P*-value	OR (95% CI)	*P*-value
Age (years)	1.023 (0.996–1.050)	0.096		
PaO_2_/FiO_2_ (mmHg)	0.995 (0.991–0.999)	0.022		
MLR	2.481 (1.710–3.601)	<0.001	2.956 (1.873–4.665)	<0.001
NLR	1.012 (0.998–1.027)	0.083	0.972 (0.950–0.994)	0.012
APACHE II score	1.097 (1.039–1.158)	0.001	1.088 (1.024–1.156)	0.007
SOFA score	1.244 (1.106–1.400)	<0.001	1.220 (1.066–1.395)	0.004
Alimentotherapy	0.434 (0.238–0.790)	0.006	2.809 (1.380–5.719)	0.004
Albumin infusion	0.411 (0.241–0.702)	0.001		
Transfusion	0.549 (0.319–0.945)	0.030		
CRRT	0.547 (0.273–1.094)	0.088		

*ARDS*, acute respiratory distress syndrome; *MLR*, monocyte-to-lymphocyte ratio; *NLR*, neutrophil-to-lymphocyte ratio; *APACHE II*, Acute Physiology and Chronic Health Evaluation II; *SOFA*, Sequential Organ Failure Assessment; *CRRT*, continuous renal replacement therapy; *OR*, odds ratio; *CI*, confidence interval

### Analysis of the efficiency of indicators in predicting 28-day mortality in patients with ARDS

**Fig. 3:**
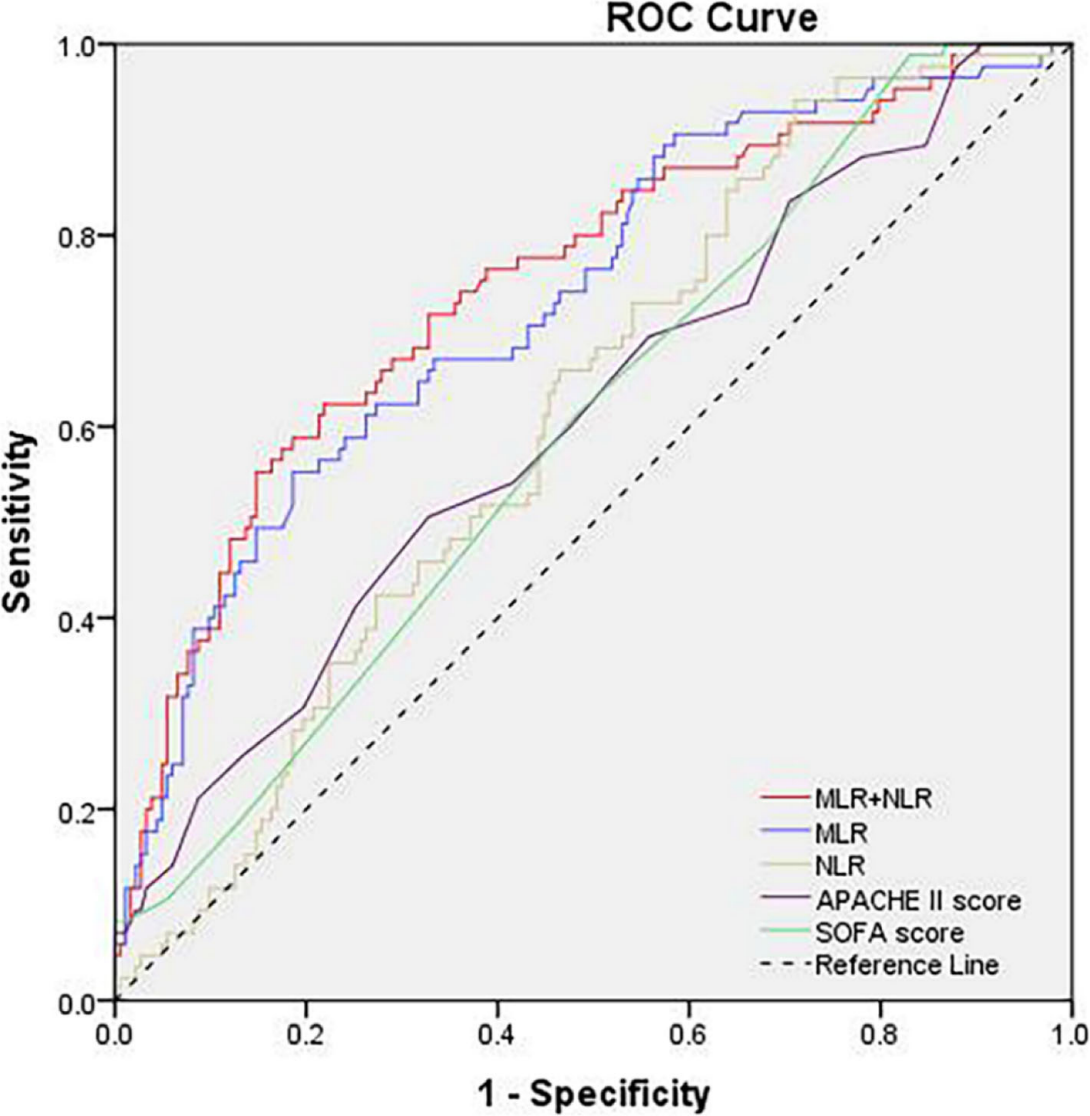
ROC curves for the APACHE II score, SOFA score, MLR, NLR, and MLR+NLR for predicting 28-day mortality in patients with ARDS. ROC, receiver operating characteristics; APACHE II, Acute Physiology and Chronic Health Evaluation II; SOFA, Sequential Organ Failure Assessment; MLR, monocyte-to-lymphocyte ratio; NLR, neutrophil-to-lymphocyte ratio; MLR+NLR, the integration parameters of MLR and NLR; ARDS, acute respiratory distress syndrome

ROC curve analysis showed that the cutoff MLR was 0.90 (55.3% sensitivity and 81.4% specificity, Table 3) to discriminate 28-day mortality, and the area under the curve (AUC) was 0.731 (95% CI 0.666–0.796, *P* <0.001, Fig. 3 and Table 3). The AUC for NLR was 0.613 (95% CI 0.544–0.681, *P*=0.003). The AUC for APACHE II score was 0.610 (95% CI 0.537–0.683, *P*=0.004). The AUC for SOFA score was 0.598 (95% CI 0.528–0.668, *P*<0.010). MLR and NLR indicators were combined for predictive efficacy analysis of 28-day mortality in ARDS patients, and its AUC was 0.750 (95% CI 0.686–0.814, *P*<0.001) (Fig. 3 and Table 3).

**Table 3:** The value of indicators in predicting 28-day mortality in patients with ARDS

	AUC	95% CI	*P*-value	Optimal cutoff value	Specificity (%)	Sensitivity (%)
APACHE II score	0.610	0.537–0.683	0.004	14.50	67.2	50.6
SOFA score	0.598	0.528–0.668	0.010	5.50	16.9	98.8
MLR	0.731	0.666–0.796	<0.001	0.90	81.4	55.3
NLR	0.613	0.544–0.681	0.003	5.27	29	94.1
MLR+NLR	0.750	0.686–0.814	<0.001	0.30	78.1	62.4

*APACHE II*, Acute Physiology and Chronic Health Evaluation II; *SOFA*, Sequential Organ Failure Assessment; *MLR*, monocyte-to-lymphocyte ratio; *NLR*, neutrophil-to-lymphocyte ratio; *MLR+NLR*: the integration parameters of MLR and NLR; *ARDS*, acute respiratory distress syndrome; *AUC*, area under the curve; *CI*, confidence interval

### Baseline characteristics of ARDS patients according to the cut-off value of MLR

Demographic, laboratory, and clinical variables, according to the optimal cutoff value of MLR 0.90, are shown in Table 4. Subjects were classified into two groups: high MLR group (MLR>0.90; *n* = 82) and low MLR group (MLR *<* 0.90, *n* = 186). There was a significant increase in 28-day mortality depending on the increasing MLR level: low MLR group 38 (20.4%), high MLR group 47 (57.3%) (*P*<0.001). As depicted in Table 4, patients with higher MLR levels had longer periods of mechanical ventilation and more likely to use interventions such as albumin infusion and alimentotherapy during hospitalization, and had higher NLR, higher Cr, and higher SOFA score. According to the optimal cutoff values of NLR (5.27), subjects were classified into two groups, and 28-day mortality in the high NLR group (NLR≥5.27, *n* =210) was significantly different from those in the low NLR group (NLR<5.27, *n* =58), (8.6%, 38.1%, respectively, *P*<0.001) (Table S2).

**Table 4:** Baseline characteristics of ARDS patients in different MLR levels

Variables	Low MLR (MLR *<* 0.90, *n* = 186)	High MLR (MLR ≥ 0.90, *n* = 82)	*P* value
Age (years)	67 (60.75–72)	68 (62–73)	0.448
Male, *n* (%)	131 (70.4)	61 (74.4)	0.507
Smoking, *n* (%)	61 (32.8)	24 (29.3)	0.567
Alcohol abuse, *n* (%)	47 (25.3)	16 (19.5)	0.306
Hypertension, *n* (%)	82 (44.1)	34 (41.5)	0.690
Diabetes mellitus, *n* (%)	50 (26.9)	24 (29.3)	0.687
Coronary artery disease, *n* (%)	39 (21)	15 (18.3)	0.615
Risk factor, *n* (%)			0.263
Pneumonia	167 (89.7)	79 (96.3)	
Aspiration	8 (4.3)	2 (2.4)	
Sepsis	5 (2.8)	1 (1.3)	
Others	6 (3.2)	0 (0)	
Types of infection, *n* (%)			0.629
Bacteria	138 (74.2)	65 (79.3)	
Virus	7 (3.8)	1 (1.2)	
Fungus	26 (14)	11 (13.4)	
Unknown	15 (8)	5 (6.1)	
PaO_2_/FiO_2_ (mmHg)	142 (101–194)	138 (87.61–184.25)	0.386
Categories of ARDS, *n* (%)			0.546
Mild	40 (21.5)	17 (20.7)	
Moderate	101 (54.3)	40 (48.8)	
Severe	45 (24.2)	25 (30.5)	
WBC, 10^9^/L	9.56 (6.86–11.75)	9.7 (6.67–11.89)	0.944
Hemoglobin, g/L	112.5 (98–129.25)	105 (91.5–124.25)	0.091
RDW, %	13.75 (12.9–15.1)	13.9 (13–14.9)	0.775
Lactate, mmol/L	2.2 (1.4–3.83)	2.6 (1.5–3.73)	0.192
Albumin, g/L	32.9 (28.9–37.7)	32.15 (28.58–37.3)	0.349
MLR	0.41 (0.26–0.59)	1.49 (1.15–2.24)	<0.001
NLR	8.45 (4.47–16.09)	20.62 (13.11–35.12)	<0.001
RDW/albumin, %/g/L	0.43 (0.36–0.51)	0.43 (0.37–0.49)	0.903
AST	50.45 (21.03–139.13)	75.25 (28.7–151.78)	0.106
ALT	54.55 (20–125.93)	62.45 (25.28–149.53)	0.465
Cr, μmol/L	94.35 (58.28–181.88)	134.7 (71.78–197.98)	0.025
BUN, mmol/L	13.28 (7.4–17.8)	13.57 (9.79–19.2)	0.154
APACHE II score	12 (9–16)	14 (10–17.25)	0.308
SOFA score	7 (6–9)	8 (7–9)	0.046
Interventions, n (%)			
Steroid^a^	129 (69.4)	55 (67.1)	0.711
Hypoglycemic	58 (31.2)	23 (28)	0.607
Alimentotherapy	113 (60.8)	66 (80.5)	0.002
Albumin infusion	82 (44.1)	55 (67.1)	0.001
Transfusion	51 (27.4)	32 (39)	0.058
CRRT	23 (12.4)	16 (19.5)	0.126
ECMO	7 (3.8)	4 (4.9)	0.672
Mechanical ventilation	109 (58.6)	50 (61)	0.715
28-day mortality, *n* (%)	38 (20.4)	47 (57.3)	<0.001
Duration of ventilation	6 (2–12)	10 (3.75–14)	0.008
Hospital length of stay	19 (12–24.25)	18.5 (12.75–25)	0.787

^a^Steroid therapy was defined as at least a dose (≥ 0.5mg/kg) of methylprednisolone during hospitalization

*ARDS*, acute respiratory distress syndrome; *WBC*, white blood cell; *RDW*, red cell distribution width; *MLR*, monocyte-to-lymphocyte ratio; *NLR*, neutrophil-to-lymphocyte ratio; *AST*, aspartate aminotransferase; *ALT*, alanine aminotransferase; *Cr*, creatinine; *BUN*, blood urea nitrogen; *APACHE II*, Acute Physiology and Chronic Health Evaluation II; *SOFA*, Sequential Organ Failure Assessment; *CRRT*, continuous renal replacement therapy; *ECMO*, extracorporeal membrane oxygenation

## Discussion

In this retrospective study, we investigated these serum inflammatory parameters (MLR, NLR, and RDW/albumin ratio) in ARDS patients and compared with preexisting indicators, such as APACHE II score and SOFA score. We found that there was an association between MLR measured within 24 h after ARDS diagnosis and the 28-day mortality in ARDS patients, in both the original and matched cohorts. MLR and NLR were significantly higher in non-survivors than in survivors and were independent risk factors of 28-day mortality. Notably, there was no difference in RDW/albumin ratio between the two groups, which was inconsistent with the results from a previous study. This study showed that the RDW/albumin ratio was significantly associated with 60-day mortality in ARDS patients [13]. In addition, we also found that the integration of MLR and NLR indicators may lead to improved prediction. Sun et al. found that the integration of MLR and NLR indicators for diagnostic analysis of severe coronavirus disease 2019 (COVID-19) achieved an AUC of 0.925 and high specificity and sensitivity [16]; this is consistent with our research.

The APACHE II score and SOFA score are commonly used in the ICU to predict the prognosis of disease; however, it has limitations in predicting the progression and disease severity of ARDS because it involves subjective measurements and complicated calculations that lead to ambiguities [17–19]. Novel inflammatory markers MLR and NLR have better kinetic patterns compared to traditional inflammatory marker, hs-CRP distribution [20]. MLR and NLR reflect two immune pathways that may be less influenced by confounding conditions and may be more predictive in assessing inflammation than assessing monocytes, lymphocytes, or neutrophils separately [21, 22]. It has been reported that an NLR >14 was associated with a shorter overall survival of ARDS patients [12]. Previous studies showed that the role of the increased MLR as a novel hematological parameter was associated with mortality in various diseases, such as solid tumors, inflammatory-related diseases, and cerebrovascular diseases [23–29]. Recent study showed a significant increase in peripheral blood combined parameters including MLR and NLR in patients with COVID-19, demonstrating the clinical importance of monitoring the changes in blood routine parameters [16, 30].

ARDS is a heterogeneous clinical condition with limited treatment options and often has fatal outcomes in critically ill patients receiving invasive mechanical ventilation [5]. Clinical and animal studies have shown that the activation of multiple inflammatory cells and the release of inflammatory mediators contributes to the development and progression of ARDS [10]. However, the relationship between poor prognosis and higher levels of MLR is not yet clear. During the initial exudative phase of lung injury, innate immune cells such as monocytes and neutrophils are recruited into the alveolar airspaces causing increase of permeability of the vascular endothelial boundary and alveolar epithelium and proteinous edema fluid to accumulate in the alveoli and interstitium [10, 31, 32]. In subsequent lung injury, macrophages in the alveoli secrete proinflammatory cytokines that help recruit circulating monocytes and neutrophils into the lungs, causing ongoing inflammation and tissue damage [33]. Monocytes have an important role in the initiation of inflammation, acting as sentinels and effectors of infection [34]. When infection occurs, monocytes were rapidly recruited to the inflammatory site, and in such case, they can develop into a wide range of terminally differentiated cells to perform multiple functions, such as enhance bactericidal activity through the production of inflammatory or anti-inflammatory mediators and cytokines and promote the renewal of tissue macrophages and dendritic cells [34]. Those may have effects on increasing MLR levels.

ARDS progresses rapidly after the initial injury; early recognition and control of inflammation are essential when treating ARDS patients. In our study, the predictive value of MLR and the difference with NLR, APACHE II score, and SOFA score were observed using the ROC curve. We found the AUC of MLR was the largest, followed by the AUC of NLR, both higher than that of APACHE II score and SOFA score. However, MLR has high specificity (81.4%) instead of low sensitivity (55.3%). The high specificity indicates that the majority of patients who died within 28 days after the onset of ARDS have higher MLR values, which would be of value in the discussion of the cause of death in ARDS. Because of low sensitivity, the value of MLR over 0.9 cannot be a good predictive indicator for 28-day mortality of the patients with ARDS. Considering that NLR has high sensitivity (94.1) instead of low specificity (29%), MLR and NLR indicators were combined for predictive efficacy analysis of 28-day mortality in ARDS patients, and it was found that the AUC reached 0.750, with higher sensitivity (62.4%) and specificity (78.1%). The majority of patients who died had higher levels of MLR, and whether this reveals some potential mechanisms for the increased mortality may be worth exploring further.

There were also several limitations to this study. First, this is a single-center retrospective study, and the sample size was not large; selection bias cannot be ruled out, so it is difficult to generalize the results to all ARDS patients. Second, the lack of clinical information is one of the limitations. We aim to conduct a multi-center study to further validate our findings in the future.

## Conclusions

In conclusion, our study showed that higher MLR values are associated with 28-day mortality of patients with ARDS, but it was not a good predictor of 28-day mortality. MLR, NLR, and other blood routine parameters have the advantages of being reliable, cost-effective, and convenient. The dynamic monitoring of blood routine parameters is of clinical importance. Future studies, particularly prospective studies with large samples, are needed to confirm these findings and explore the potential mechanisms of MLR in ARDS patients.

## Supplementary Information

**Additional file 1.** Table S1.Univariate logistic regression analysis of 28-day mortality prediction for patients with ARDS. Table S2. Baseline characteristics of ARDS patients in different NLR level. Table S3. Baseline characteristics of ARDS patients in different RDW/Albumin ratio level.

## Data Availability

The datasets used and/or analyzed during the current study are available from the corresponding author on reasonable request.

## Abbreviations

ARDS: Acute respiratory distress syndrome
WBC: White blood cell
RDW: Red cell distribution width
MLR: Monocyte-to-lymphocyte ratio
NLR: Neutrophil-to-lymphocyte ratio
PSM: Propensity score matching
COVID-19: Coronavirus disease 2019
AST: Aspartate aminotransferase
ALT: Alanine aminotransferase
Cr: Creatinine
BUN: Blood urea nitrogen
CRRT: Continuous renal replacement therapy
ECMO: Extracorporeal membrane oxygenation
APACHE II: Acute Physiology and Chronic Health Evaluation II
SOFA: Sequential Organ Failure Assessment
OR: Odds ratio
CI: Confidence interval
ROC: Receiver operating characteristics
AUC: Area under the curve
