# Supplementary material for: Monocyte-to-lymphocyte ratio is associated with 28-day mortality in patients with acute respiratory distress syndrome: a retrospective study
Source: J Intensive Care. 2021 Aug 6;9:49. doi: 10.1186/s40560-021-00564-6 (PMC8342981; doi:10.1186/s40560-021-00564-6)
Supplement: Supplementary file 1 — Additional file 1. Table S1.Univariate logistic regression analysis of 28-day mortality prediction for patients with ARDS. Table S2. Baseline characteristics of ARDS patients in different NLR level. Table S3. Baseline characteristics of ARDS patients in different RDW/Albumin ratio level. [file 40560_2021_564_MOESM1_ESM.docx]

TableS1.Univariate logistic regression analysis of 28-day mortality prediction for patients with ARDS.

| Variables | Original cohort | |
| --- | --- | --- |
|  | OR (95% CI) | P-value |
| Age (years) | 1.023(0.996-1.050) | 0.096 |
| Male, n (%) | 1.172(0.667-2.061) | 0.581 |
| Smoking, n (%) | 1.171(0.669-2.048) | 0.581 |
| Alcohol abuse, n (%) | 1.214(0.653-2.255) | 0.540 |
| Hypertension, n (%) | 1.511(0.891-2.563) | 0.126 |
| Diabetes mellitus, n (%) | 1.137(0.635-2.034) | 0.666 |
| Coronary Artery Disease, n (%) | 0.912(0.483-1.721) | 0.775 |
| Risk factor, n (%) |  |  |
| Pneumonia | Reference | |
| Aspiration | 0.528(0.110-2.546) | 0.427 |
| Sepsis | 2.114(0.417-10.709) | 0.366 |
| Others | 0.423(0.049-3.679) | 0.435 |
| Types of Infection, n (%) |  |  |
| Virus | Reference | |
| Bacteria | 1.381(0.271-7.032) | 0.697 |
| Fungus | 1.773(0.311-10.110) | 0.519 |
| Unknow | 1.125(0.176-7.191) | 0.901 |
| PaO2/FiO2 (mmHg) | 0.995(0.991-0.999) | 0.022 |
| WBC, 10^9^ /L | 0.963(0.901-1.029) | 0.262 |
| Hemoglobin, g/L | 0.995(0.983-1.007) | 0.389 |
| RDW, % | 0.931(0.807-1.076) | 0.333 |
| Lactate, mmol/L | 1.105(0.961-1.270) | 0.162 |
| Albumin, g/L | 0.985(0.947-1.025) | 0.460 |
| MLR | 2.481(1.710-3.601) | <0.001 |
| NLR | 1.012(0.998-1.027) | 0.083 |
| RDW/Albumin, %/g/L | 1.023(0.113-9.269) | 0.984 |
| AST | 1.001(0.998-1.004) | 0.561 |
| ALT | 0.999(0.996-1.002) | 0.442 |
| Cr, μ mol/L | 1.001(0.999-1.003) | 0.368 |
| BUN, mmol/L | 1.017(0.984-1.051) | 0.313 |
| APACHE II score | 1.097(1.039-1.158) | 0.001 |
| SOFA score | 1.244(1.106-1.400) | <0.001 |
| Interventions, n (%) |  |  |
| Steroid^a^ | 0.682(0.384-1.210) | 0.190 |
| Hypoglycemic | 1.058(0.603-1.858) | 0.844 |
| Alimentotherapy | 0.434(0.238-0.790) | 0.006 |
| Albumin infusion | 0.411(0.241-0.702) | 0.001 |
| Transfusion | 0.549(0.319-0.945) | 0.030 |
| CRRT | 0.547(0.273-1.094) | 0.088 |
| ECMO | 0.542(0.161-1.829) | 0.324 |
| Mechanical ventilation | 0.960(0.568-1.622) | 0.879 |
| Duration of ventilation | 1.006(0.970-1.044) | 0.730 |

ARDS, acute respiratory distress syndrome; MLR, monocyte to lymphocyte ratio; NLR, neutrophil to lymphocyte ratio; APACHE II, Acute Physiology and Chronic Health Evaluation II; SOFA, sequential organ failure assessment; CRRT, continuous renal replacement therapy; OR, odds ratio; CI, confidence interval; ECMO: extracorporeal membrane oxygenation.

TableS2. Baseline characteristics of ARDS patients in different NLR level.

| Variables | Low NLR  (NLR<5.27, n =58) | High NLR  (NLR≥5.27, n =210) | P value |
| --- | --- | --- | --- |
| Age (years) | 65(54.5-73.25) | 67(62-73) | 0.314 |
| Male, n (%) | 40(69) | 152(72.4) | 0.609 |
| Smoking, n (%) | 12(20.7) | 73(34.8) | 0.041 |
| Alcohol abuse, n (%) | 10(17.2) | 53(25.2) | 0.204 |
| Hypertension, n (%) | 30(51.7) | 86(41) | 0.143 |
| Diabetes mellitus, n (%) | 23(39.7) | 51(24.3) | 0.020 |
| Coronary Artery Disease, n (%) | 13(22.4) | 41(19.5) | 0.627 |
| Risk factor, n (%) |  |  | 0.160 |
| Pneumonia | 50(86.2) | 196(93.3) |  |
| Aspiration | 4(6.9) | 6(2.9) |  |
| Sepsis | 1(1.7) | 5(2.4) |  |
| Others | 3(5.2) | 3(1.4) |  |
| Types of Infection, n (%) |  |  | 0.078 |
| Bacteria | 44(75.9) | 159(75.7) |  |
| Virus | 0(0) | 8(3.8) |  |
| Fungus | 6(10.3) | 31(14.8) |  |
| Unknow | 8(13.8) | 12(5.7) |  |
| PaO2/FiO2 (mmHg) | 156.5(111-226.29) | 140.5(95.94-183.54) | 0.031 |
| Categories of ARDS, n (%) |  |  | <0.001 |
| Mild | 25(43.1) | 32(15.2) |  |
| Moderate | 24(41.4) | 117(55.7) |  |
| Severe | 9(15.5) | 61(29.1) |  |
| WBC, 10^9^ /L | 9.69(5.95-12.13) | 9.57(6.95-11.62) | 0.947 |
| Hemoglobin, g/L | 116(100.5-127.5) | 109.5(95.75-126) | 0.314 |
| RDW, % | 14.1(13-15.38) | 13.7(12.9-14.9) | 0.190 |
| Lactate, mmol/L | 1.95(1.1-4.05) | 2.6(1.5-3.7) | 0.119 |
| Albumin, g/L | 32.95(29.4-38.38) | 32.45(28.78-37.6) | 0.401 |
| MLR | 0.28(0.18-0.37) | 0.68(0.43-1.24) | <0.001 |
| NLR | 3.34(1.85-4.26) | 14.97(10.06-24.04) | <0.001 |
| RDW/Albumin, %/g/L | 0.43(0.36-0.53) | 0.43(0.37-0.5) | 0.870 |
| AST | 32.3(18.3-124.68) | 71(26.68-149.5) | 0.020 |
| ALT | 30(16.4-99.9) | 62(25.5-140.88) | 0.012 |
| Cr, μ mol/L | 75.25(50.45-177.45) | 109.5(62.85-185.35) | 0.088 |
| BUN, mmol/L | 12.55(5.38-18.8) | 13.49(8.78-18.13) | 0.315 |
| APACHE II score | 13(8-16) | 13(10-17) | 0.156 |
| SOFA score | 7(6-8) | 8(6-9) | 0.001 |
| Interventions, n (%) |  |  |  |
| Steroid^a^ | 33(56.9) | 151(71.9) | 0.029 |
| Hypoglycemic | 27(46.6) | 54(25.7) | 0.002 |
| Alimentotherapy | 35(60.3) | 145(69) | 0.212 |
| Albumin infusion | 23(39.7) | 114(54.3) | 0.048 |
| Transfusion | 12(20.7) | 71(33.8) | 0.056 |
| CRRT | 8(13.8) | 31(14.8) | 0.853 |
| ECMO | 2(3.4) | 9(4.3) | 0.776 |
| Mechanical ventilation | 39(67.2) | 120(57.1) | 0.166 |
| 28-day mortality, n (%) | 5(8.6) | 80(38.1) | <0.001 |
| Duration of ventilation | 7.5(1.75-14) | 7(3-13) | 0.941 |
| Hospital length of stay | 23(12.75-28) | 18(12-24) | 0.039 |

^a^ Steroid therapy was defined as at least a dose (≥ 0.5mg/kg) of methylprednisolone during hospitalization. ARDS, acute respiratory distress syndrome; WBC, white blood cell; RDW: red cell distribution width; MLR, monocytes to lymphocytes ratio; NLR, neutrophil-to-lymphocyte ratio; AST, aspartate aminotransferase; ALT, alanine aminotransferase; Cr, creatinine; BUN, blood urea nitrogen; APACHE II, Acute Physiology and Chronic Health Evaluation II; SOFA, sequential organ failure assessment; CRRT, continuous renal replacement therapy; ECMO: extracorporeal membrane oxygenation.

TableS3. Baseline characteristics of ARDS patients in different RDW/Albumin ratio level.

| Variables | Low RDW/Albumin ratio  (<0.41, n =103) | High RDW/Albumin ratio  (≥0.41, n =165) | P value |
| --- | --- | --- | --- |
| Age (years) | 66(59-72) | 68(62-73) | 0.123 |
| Male, n (%) | 77(74.8) | 115(69.7) | 0.371 |
| Smoking, n (%) | 32(31.1) | 53(32.1) | 0.857 |
| Alcohol abuse, n (%) | 21(20.4) | 42(25.5) | 0.341 |
| Hypertension, n (%) | 51(49.5) | 31(18.8) | 0.073 |
| Diabetes mellitus, n (%) | 36(35) | 38(23) | 0.034 |
| Coronary Artery Disease, n (%) | 20(19.4) | 34(20.6) | 0.813 |
| Risk factor, n (%) |  |  | 0.550 |
| Pneumonia | 94(91.3) | 152(92.1) |  |
| Aspiration | 5(4.8) | 5(3) |  |
| Sepsis | 1(0.9) | 5(3) |  |
| Others | 3(3) | 3(1.9) |  |
| Types of Infection, n (%) |  |  | 0.785 |
| Bacteria | 77(74.8) | 126(76.4) |  |
| Virus | 2(1.9) | 6(3.6) |  |
| Fungus | 15(14.6) | 22(13.3) |  |
| Unknow | 9(8.7) | 11(6.7) |  |
| PaO2/FiO2 (mmHg) | 160(109.51-220) | 136(100-180.5) | 0.070 |
| Categories of ARDS, n (%) |  |  | 0.002 |
| Mild | 33(32) | 24(14.5) |  |
| Moderate | 49(47.6) | 92(55.8) |  |
| Severe | 21(20.4) | 49(29.7) |  |
| WBC, 10^9^ /L | 9.49(5.95-11.68) | 9.7(7.46-11.8) | 0.374 |
| Hemoglobin, g/L | 119(101-134) | 108(92-121.5) | <0.001 |
| RDW, % | 13.2(12.6-14) | 14.5(13.3-15.8) | <0.001 |
| Lactate, mmol/L | 2.5(1.4-4.1) | 2.5(1.4-3.7) | 0.825 |
| Albumin, g/L | 38.1(35.5-41.7) | 29.9(26-32.9) | <0.001 |
| MLR | 0.63(0.36-1.08) | 0.53(0.28-1.07) | 0.230 |
| NLR | 10.9(6.1-17.46) | 13.17(6.3-21.99) | 0.192 |
| RDW/Albumin, %/g/L | 0.36(0.32-0.38) | 0.47(0.43-0.54) | <0.001 |
| AST | 41.5(18.6-137) | 78.4(26.9-153.65) | 0.017 |
| ALT | 48.4(17.3-108.1) | 62(21.1-142.3) | 0.081 |
| Cr, μ mol/L | 86.4(56.7-158.9) | 117.1(66.85-191.75) | 0.023 |
| BUN, mmol/L | 13.35(8.2-17.7) | 13.43(7.85-19) | 0.461 |
| APACHE II score | 13(9-17) | 13(10-16) | 0.979 |
| SOFA score | 8(6-9) | 8(6.5-9) | 0.340 |
| Interventions, n (%) |  |  |  |
| Steroid^a^ | 67(65) | 117(70.9) | 0.314 |
| Hypoglycemic | 41(39.8) | 40(24.2) | 0.007 |
| Alimentotherapy | 73(70.9) | 106(64.2) | 0.262 |
| Albumin infusion | 52(50.5) | 85(51.5) | 0.870 |
| Transfusion | 24(23.3) | 59(35.8) | 0.032 |
| CRRT | 20(19.4) | 19(11.5) | 0.074 |
| ECMO | 4(3.9) | 7(4.2) | 0.885 |
| Mechanical ventilation | 64(62.1) | 95(57.6) | 0.460 |
| 28-day mortality, n (%) | 0(0) | 85(51.5) | <0.001 |
| Duration of ventilation | 7(2-13) | 7(3-13) | 0.909 |
| Hospital length of stay | 21(13-27) | 17(12-24) | 0.050 |

^a^ Steroid therapy was defined as at least a dose (≥ 0.5mg/kg) of methylprednisolone during hospitalization. ARDS, acute respiratory distress syndrome; WBC, white blood cell; RDW: red cell distribution width; MLR, monocytes to lymphocytes ratio; NLR, neutrophil-to-lymphocyte ratio; AST, aspartate aminotransferase; ALT, alanine aminotransferase; Cr, creatinine; BUN, blood urea nitrogen; APACHE II, Acute Physiology and Chronic Health Evaluation II; SOFA, sequential organ failure assessment; CRRT, continuous renal replacement therapy; ECMO: extracorporeal membrane oxygenation.
